# Modification of a loop sequence between α-helices 6 and 7 of virus capsid (CA) protein in a human immunodeficiency virus type 1 (HIV-1) derivative that has simian immunodeficiency virus (SIVmac239) *vif *and CA α-helices 4 and 5 loop improves replication in cynomolgus monkey cells

**DOI:** 10.1186/1742-4690-6-70

**Published:** 2009-08-03

**Authors:** Ayumu Kuroishi, Akatsuki Saito, Yasuhiro Shingai, Tatsuo Shioda, Masako Nomaguchi, Akio Adachi, Hirofumi Akari, Emi E Nakayama

**Affiliations:** 1 Department of Viral Infections, Research Institute for Microbial Diseases, Osaka University, Osaka 565-0871, Japan; 2 Tsukuba Primate Research Center, National Institute of Biomedical Innovation, Ibaraki 305-0843, Japan; 3 Department of Virology, Institute of Health Biosciences, University of Tokushima Graduate School, Tokushima 770-8503, Japan

## Abstract

**Background:**

Human immunodeficiency virus type 1 (HIV-1) productively infects only humans and chimpanzees but not cynomolgus or rhesus monkeys while simian immunodeficiency virus isolated from macaque (SIVmac) readily establishes infection in those monkeys. Several HIV-1 and SIVmac chimeric viruses have been constructed in order to develop an animal model for HIV-1 infection. Construction of an HIV-1 derivative which contains sequences of a SIVmac239 loop between α-helices 4 and 5 (L4/5) of capsid protein (CA) and the entire SIVmac239 *vif *gene was previously reported. Although this chimeric virus could grow in cynomolgus monkey cells, it did so much more slowly than did SIVmac. It was also reported that intrinsic TRIM5α restricts the post-entry step of HIV-1 replication in rhesus and cynomolgus monkey cells, and we previously demonstrated that a single amino acid in a loop between α-helices 6 and 7 (L6/7) of HIV type 2 (HIV-2) CA determines the susceptibility of HIV-2 to cynomolgus monkey TRIM5α.

**Results:**

In the study presented here, we replaced L6/7 of HIV-1 CA in addition to L4/5 and *vif *with the corresponding segments of SIVmac. The resultant HIV-1 derivatives showed enhanced replication capability in established T cell lines as well as in CD8+ cell-depleted primary peripheral blood mononuclear cells from cynomolgus monkey. Compared with the wild type HIV-1 particles, the viral particles produced from a chimeric HIV-1 genome with those two SIVmac loops were less able to saturate the intrinsic restriction in rhesus monkey cells.

**Conclusion:**

We have succeeded in making the replication of simian-tropic HIV-1 in cynomolgus monkey cells more efficient by introducing into HIV-1 the L6/7 CA loop from SIVmac. It would be of interest to determine whether HIV-1 derivatives with SIVmac CA L4/5 and L6/7 can establish infection of cynomolgus monkeys *in vivo*.

## Background

Human immunodeficiency virus type 1 (HIV-1) productively infects only humans and chimpanzees but not Old World monkeys (OWM) such as cynomolgus (CM) and rhesus (Rh) monkeys [1]. Unlike the simian immunodeficiency virus isolated from macaques (SIVmac), HIV-1 replication is blocked early after viral entry, before the establishment of a provirus in OWM cells [1-3]. This restricted host range of HIV-1 has greatly hampered its use in animal experiments and has caused difficulties for developing prophylactic vaccines and understanding HIV-1 pathogenesis. In order to establish a monkey model of HIV-1/AIDS, various chimeric viral genomes between SIVmac and HIV-1 (SHIV) have been constructed and tested for their replicative capabilities in simian cells. The first SHIV was generated on a genetic background of SIVmac with HIV-1 *tat*, *rev*, *vpu*, and *env *genes [4]. Although such a SHIV is useful for the analysis of humoral immune responses against the Env protein [5-7], SHIVs containing other HIV-1 structural proteins, especially the Gag-Pol protein, have become highly desirable, since cellular immune response against Gag is generally believed to be important for disease control [8-10].

In recent years, several host factors involved in HIV-1 restriction in OWM cells have been identified. ApoB mRNA editing catalytic subunit (APOBEC) 3 G modifies the minus strand viral DNA during reverse transcription, resulting in an impairment of viral replication [11-13]. This activity could be counteracted with the viral protein Vif [14-17]. Although HIV-1 Vif can potently suppress human APOBEC3G, it is not effective against Rh APOBEC3G, which explains at least partly why HIV-1 replication is restricted in monkey cells. It is well known that Cyclophilin A (CypA) binds directly to the exposed loop between α-helices 4 and 5 (L4/5) of HIV-1 capsid protein (CA), but not to the SIVmac CA. Several studies have found that CypA augments HIV-1 infection in human cells but inhibits its replication in OWM cells [18-20]. A construction of a SHIV with a minimal segment of SIVmac was reported recently by Kamada et al. [21]. This SHIV was designed to evade the restrictions mediated by APOBEC3G and CypA in OWM cells and contains the 7-aa segment corresponding to the L4/5 of CA and the entire *vif *of SIVmac. The SHIV was found to be able to replicate in primary CD4+ T cells from pig-tailed monkey as well as in the CM HSC-F T cell line. Both in HSC-F and in primary CD4+ T cells, this chimeric virus grew to lower titers than did SIVmac [21]; and when inoculated into pig-tailed monkeys, this SHIV did not cause CD4+ T cell depletion or any clinical symptoms in the inoculated animals [22]. Another SHIV, stHIV-1 (a virus carrying 202 amino acid residues of SIVmac CA and *vif *generated by Hatziioannou et al.) could replicate efficiently in Rh cells [23]. However, long-term passaging in Rh cells was necessary to generate an efficiently replicating stHIV-1, and this adapted virus has not yet been fully characterized; so it may be that further modifications of the viral genome are necessary for optimal replication of HIV-1 genomes in OWM cells.

TRIM5α, a member of the tripartite motif (TRIM) family proteins, was identified in 2004 as another intrinsic restriction factor of HIV-1 in OWM cells [24]. Rh and CM TRIM5α were found to restrict HIV-1 but not SIVmac [25,26]. TRIM5α recognizes the multimerized CA of an incoming virus by its α-isoform specific SPRY domain [27-29] and is believed to be involved in innate immunity to control retroviral infection [30]. Previously, Ylinen et al. mapped one of the determinants of TRIM5α sensitivity in L4/5 of HIV type 2 (HIV-2) CA [31]. In addition, we identified a single amino acid of the surface-exposed loop between α-helices 6 and 7 (L6/7) of HIV-2 CA as a determinant of the susceptibility of HIV-2 to CM TRIM5α [32]. We hypothesized that the L6/7 of HIV-1 CA also determines susceptibility to CM TRIM5α. Here, we investigated whether an additional replacement of L6/7 of HIV-1 CA with that of SIVmac would enhance the replication capability of a SHIV genome in established T cell line HSC-F and in CD8+ cell depleted peripheral blood mononuclear cells (PBMCs) from CMs.

## Materials and methods

### DNA constructions

The HIV-1 derivatives were constructed on a background of infectious molecular clone NL4-3 [33]. NL-ScaVR, a virus containing SIVmac239 L4/5 and the entire vif gene, was constructed according to the procedure described by Kamada et al. [21]. A single amino acid His (H) at the 120th position of NL-ScaVR CA was replaced with Gln (Q) by means of site-directed mutagenesis with the PCR-mediated overlap primer extension method [34], and the resultant construct was designated NL-ScaVRA1. The L6/7 of CA (HNPPIP) of NL-SVR, NL-ScaVR, or NL-DT5R was also replaced with the corresponding segments of SIVmac239 CA (RQQNPIP) by means of site-directed mutagenesis, and the resultant constructs were designated NL-SVR6/7S, NL-ScaVR6/7S, or NL-DT5R6/7S, respectively. The BssHII-ApaI fragment of NL-ScaVR, NL-SVR6/7S, or NL-ScaVR6/7S, which corresponds to matrix (MA) and CA, was transferred to env deleted NL4-3 (NL-Nhe) to generate the env (-) version of each of the constructs.

### Cells and Virus propagation

The 293 T (human kidney), LLC-MK2 (Rh kidney), and TK-ts13 (hamster kidney) adherent cell lines were cultured in Dulbecco's modified Eagle medium supplemented with 10% heat-inactivated FBS. The CD4+ CXCR4+ CM T cell line HSC-F [35] was maintained in RPMI 1640 medium containing 10% FBS. Virus stocks were prepared by transfection of 293 T cells with HIV-1 NL4-3 derivatives using the calcium phosphate co-precipitation method. Viral titers were measured with the p24 or p27 RetroTek antigen ELISA kit (ZeptoMetrix, Buffalo, NY), and viral reverse transcriptase (RT) was quantified with the Reverse Transcriptase Assay kit (Roche Applied Science, Mannheim Germany).

### Green fluorescence protein (GFP) vector

The HIV-1 vector expressing GFP was prepared as described previously [36,37]. To construct the HIV-1-WT-GFP and HIV-1-L4/5S-GFP vector, we replaced the Eco RI-Apa I fragment corresponding to MA and CA of the pMDLg/p.RRE packaging vector with those fragments from NL4-3 and NL-ScaVR, respectively. The GFP viruses were prepared from 293 T cells in a 15-cm dish by co-transfection with a combination of 24 μg of pMDLg/p.RRE derivatives, 36 μg of CS-CDF-CG-PRE (GFP encoding viral genomic plasmid), 10 μg of pMD.G (vesicular stomatitis virus glycoprotein (VSV-G) expressing plasmid), and 10 μg of pRSV-Rev (Rev expressing plasmid). Forty-eight hours after transfection, the culture supernatants were collected and used for infection.

### Viral infections

3 × 10^5 ^MT4 or HSC-F cells were infected with 20 ng of p24 of NL4-3, NL-ScaV, NL-ScaVR, NL-ScaVR6/7S, NL-DT5R, or NL-DT5R6/7S. The culture supernatants were collected periodically, and p24 levels were measured with an ELISA kit.

### Particle purification and Western blotting

The culture supernatant of 293 T cells transfected with plasmids encoding HIV-1 NL4-3 derivatives was clarified by means of low speed centrifugation. Nine ml of the resultant supernatants were layered onto a 2 ml cushion of 20% sucrose (made in PBS) and centrifuged at 35,000 rpm for 2 h in a Beckman SW41 rotor. After centrifugation, the virion pellets were resuspended in PBS, and p24 antigen concentrations were measured with ELISA. SDS-polyacrylamide gel electrophoresis was applied to 120 ng of p24 of HIV-1 derivatives, and virion-associated proteins were transferred to a PVDF membrane. CA and CypA proteins were visualized with the anti-p24 antibody (Biodesign International, Saco, ME) and the anti-CypA antibody (Affinity BioReagents, Golden, CO), respectively.

### Saturation assay

HIV-1 derivatives or SIVmac particles were prepared by transfecting each of the env-deleted HIV-1 NL4-3 derivatives or SIVmac plasmids with a plasmid encoding VSV-G into 293 T cells, and culture supernatants were collected two days after transfection. One day before infection, Rh LLC-MK2 and hamster TK-ts13 were plated at a density of 5 × 10^4 ^cells per well in a 24-well plate. Prior to GFP virus infection, the cells were pretreated for 2 hours with 200 ng of p24 of each of the HIV-1 or SIVmac particles pseudotyped with VSV-G. Immediately after the pre-treatment, the cells were washed and infected with the HIV-1-WT-GFP or HIV-1-L4/5S-GFP virus. Two hours after infection, the inoculated GFP viruses were washed, and the cells were cultivated in fresh media. Two days after infection, the cells were fixed by formaldehyde, and GFP expressing cells were counted with a flowcytometer. To suppress endogenous TRIM5α activity, the cells were first infected with Sendai (SeV) expressing TRIM5 lacking the SPRY domain at a multiplicity of infection of 10 plaque forming units per cell. Sixteen hours after SeV infection, the cells were treated with 200 ng of p24 of the particles and then infected with the HIV-1-L4/5S-GFP vector as described above.

### Preparation of CD8-depleted CM PBMCs and viral infection

CM PBMCs were suspended in RPMI medium 1640 supplemented with 10% (vol/vol) FBS, and the CD8+ cells were removed with a magnetic bead system (Miltenyi Biotec, Auburn, CA) and stimulated for 1 day with 1 μg/ml of PHA-L (Sigma, St. Louis. MO). For prolonged stimulation, CD8-depleted CM PBMCs were first stimulated with 1 μg/ml of PHA-L for 2 days and then with human IL2 100 U/ml for 2 more days. 3 × 10^5 ^cells were then inoculated with 200 ng of p24 of NL-DT5R, NL-DT5R6/7S or with 200 ng of p27 of SIVmac239 and incubated at 37°C in a medium containing 100 U/ml of human IL2. The culture supernatants were collected periodically, and the levels of p24 or p27 were measured with an antigen capture assay (Advanced BioScience Laboratories, Kensington, MD)

## Results

### Construction and characterization of HIV-1 molecular clones containing CA and Vif sequences from SIVmac239

**Figure 1:**
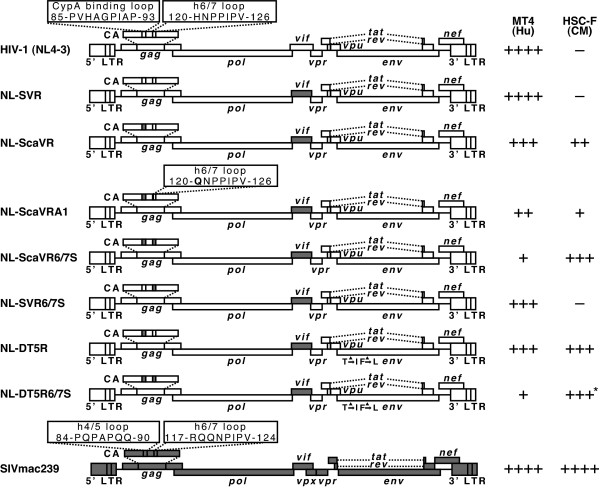
**Structure of the chimeric HIV-1/SIVmac clones and a summary of their replication capabilities**. White bars denote HIV-1 (NL4-3) and gray bars SIVmac239 sequences. ++++, +++, ++, +, and -denote the peak titer of virus growth in human (Hu) and cynomolgus monkey (CM) cells, respectively, to more than 1000 ng/ml, 100–1000 ng/ml, 10–100 ng/ml, 1–10 ng/ml, and less than 1 ng/ml concentration of capsid (CA) protein in the culture supernatants. * denotes that NL-DT5R6/7S replicated faster in HSC-F than did the parental NL-DT5R (see Fig. 2C).

**Figure 2:**
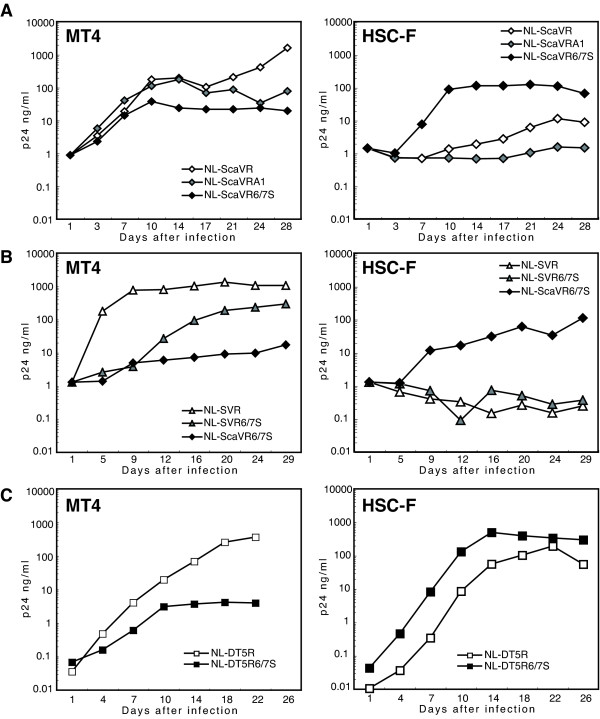
**Replication properties of HIV-1 derivatives**. Equal amounts of (A) NL-ScaVR (white diamonds: virus with SIVmac L4/5 and *vif*), and NL-ScaVRA1 (gray diamonds: virus with additional replacement of the 120th amino acid His with Gln in NL-ScaVR), and NL-ScaVR6/7S (black diamonds: virus with SIVmac L4/5, L6/7, and *vif*) (B) NL-SVR, NL-ScaVR6/7S, and NL-SVRS6/7S (gray diamonds: virus with SIVmac L6/7 and *vif*), and (C) NL-DT5R (white squares) and NL-DT5R6/7S (black squares), were inoculated into human MT4 or CM HSC-F cells, and culture supernatants were collected periodically. p24 antigen levels were measured by ELISA.

Several proviral DNA constructs have been generated to counteract the restriction of HIV-1 replication in CM T cell line HSC-F [38] (Fig. 1). We first generated NL-SVR and NL-ScaVR according to the procedure described by Kamada et al. [21]. NL-ScaVR, a virus with SIVmac239 L4/5 CA and *vif*, could replicate slowly in HSC-F and replicated well in MT4 as previously reported (Fig. 2A). We recently discovered that the 120th amino acid of CA affected the sensitivity of HIV-2 to CM TRIM5α [32]. We, therefore, introduced an additional amino acid substitution, His to Gln, at this position in NL-ScaVR. The resultant virus was designated NL-ScaVRA1; but this virus unexpectedly showed less efficient replication than did the parental NL-ScaVR in both MT4 and HSC-F cells (Fig. 2A), probably due to a reduced viral fitness created by this mutation. We, therefore, replaced the entire L6/7 CA of NL-ScaVR (HNPPIP) with the corresponding loop from SIVmac239 (RQQNPIP), and the resultant virus was designated NL-ScaVR6/7S. The amount of RT per 1 ng of CA of NL-ScaVR (0.083 ng) was comparable to that of NL-ScaVR6/7S (0.081 ng), indicating that the replacement of L6/7 in HIV-1 with the corresponding loop of SIVmac did not affect the reactivity of CA antigen. Although NL-ScaVR6/7S grew slightly slower in MT4 cells, it could replicate more efficiently in HSC-F cells than the parental NL-ScaVR could (Fig. 2A). Similar results were obtained when we inoculated 20 ng of RT equivalent of NL-ScaVR or NL-ScaVR6/7S into HSC-F cells and measured the periodic RT production in culture supernatants (data not shown). These findings demonstrated that L6/7 CA of SIVmac improved the replication in CM cells of an HIV-1 derivative that already contained a SIVmac L4/5 and *vif*. We then generated NL-SVR6/7S, in which the L4/5 sequence was from HIV-1, but the L6/7 and *vif *came from SIVmac. NL-SVR6/7S showed better replication than NL-ScaVR6/7S in MT4 cells, but lost its replicative capability in HSC-F cells (Fig. 2B). NL-SVR, a virus with SIVmac *vif*, could replicate in MT4, but failed to do so in HSC-F (Fig. 2B). These results indicated that both L4/5 and L6/7 of SIVmac are required for efficient replication in HSC-F.

We then introduced SIVmac L6/7 into NL-DT5R, a molecularly cloned virus with two nonsynonymous changes in the *env *gene gained during long-term passages of NL-ScaVR in HSC-F cells [21]. The resultant virus was designated NL-DT5R6/7S. Although the peak titer of NL-DT5R6/7S was almost the same as that of NL-DT5R, NL-DT5R6/7S could replicate faster in HSC-F than the parental NL-DT5R (Fig. 2C). This finding confirmed that SIVmac L6/7 CA sequence improved the replication in CM cells of HIV-1 derivatives that contained SIVmac L4/5 and *vif*. The finding suggested that HIV-1 L6/7 and L4/5 CA sequences are important for intrinsic restriction in CM cells.

### CypA incorporation into virus particles was not affected by replacement of HIV-1 L6/7 with that of SIVmac

**Figure 3:**
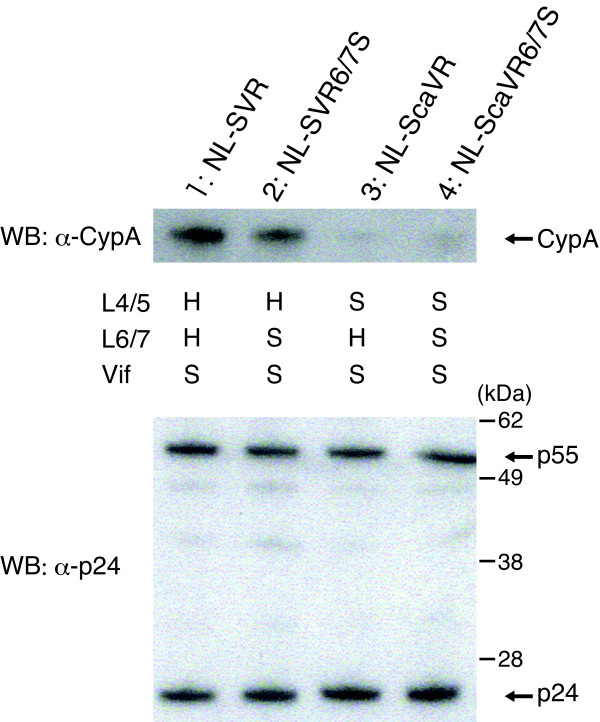
**Western blot analysis of CA and CypA in particles of HIV-1 derivatives**. The viral particles of NL-SVR (lane 1), NL-SVR6/7S (lane 2), NL-ScaVR (lane 3) and NL-ScaVR6/7S (lane 4) were purified by ultracentrifugation through a 20% sucrose cushion. CypA (upper panel) and p24 and p55 proteins (lower panel) were visualized by Western blotting (WB) using anti-CypA and anti-p24 antibody, respectively. "H" and "S" denote the amino acid sequences derived from HIV-1 and SIVmac, respectively.

Several studies have demonstrated that CypA augments HIV-1 infection in human cells [39], but inhibits its replication in OWM cells [18-20]. CypA was packaged in HIV-1 but not in SIVmac virus particles. To determine whether the replacement of HIV-1 L6/7 with that of SIVmac affects CypA binding of HIV-1 CA, we performed Western blot analysis of viral particles from HIV-1 derivatives. As shown in Fig. 3 (upper panel), CypA proteins were clearly detected in the NL-SVR particles (lane 1) but not in those of NL-ScaVR (lane 3), thus confirming that the L4/5 sequence of HIV-1 but not of SIVmac is required for CypA incorporation into viral particles. CypA proteins were detected in NL-SVR6/7S (lane 2) but not in NL-ScaVR6/7S (lane 4), indicating that the additional replacement of HIV-1 L6/7 with that of SIVmac had little effect on CypA incorporation. This finding suggests that the effect of L6/7 replacement on viral growth was independent from CypA binding of HIV-1 CA. When we used anti-p24 antibody (Fig. 3, lower panel), p55 Gag precursors and p24 proteins were clearly detected. There were no differences in the amount of p24 or the ratio of p24 to p55 among the four HIV-1 derivatives, indicating that the HIV-1 Gag precursor proteins with SIVmac L4/5 and L6/7 were processed normally by the viral protease.

### Replacement of both L4/5 and L6/7 of HIV-1 CA with the corresponding loops from SIVmac impaired the CA binding activity of TRIM5α in Rh cells

**Figure 4:**
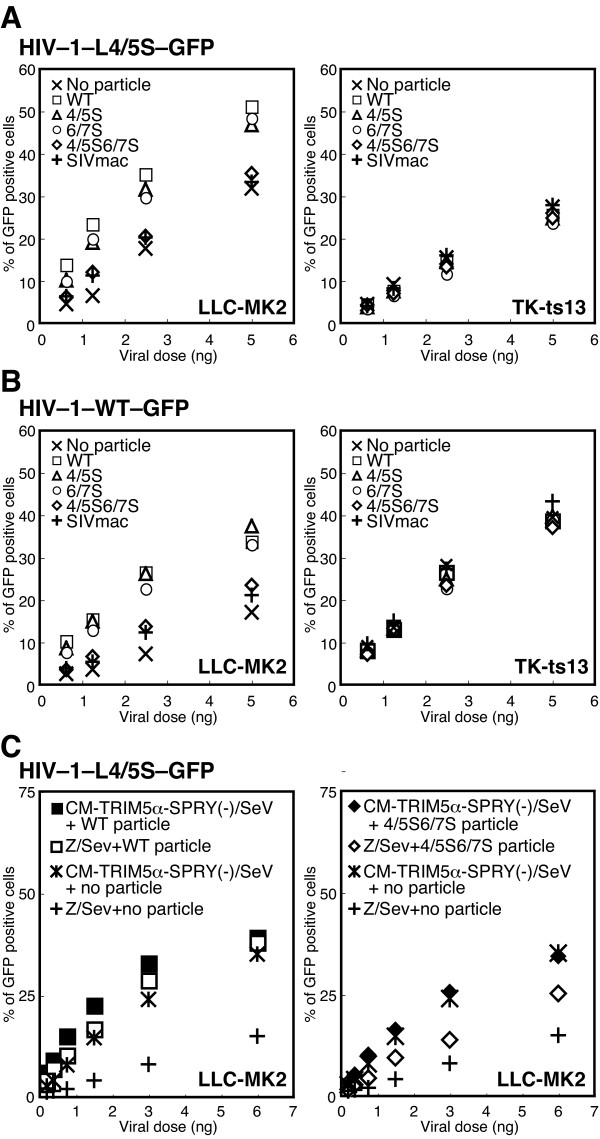
**Saturation of intrinsic antiviral factors resulting from inoculation of high dose of virus particles**. (A) Rhesus LLC-MK2 cells or hamster TK-ts13 cells were pre-treated with equal amounts of VSV-G pseudotyped particles with WT HIV-1 (white squares: Wt), with SIVmac L4/5 (white triangles: 4/5S), with SIVmac L6/7 (white circles: 6/7S), with SIVmac L4/5 and L6/7 (white diamonds: 4/5S6/7S), with SIVmac239 (pluses: SIVmac) or none (crosses) for 2 hours. The cells were then infected with the GFP expressing HIV-1 vector carrying SIVmac L4/5 (A: HIV-1-L4/5S-GFP) or GFP expressing HIV-1 vector with WT capsid (B: HIV-1-WT-GFP). Representative data of four independent experiments are shown. (C) Saturation activities were assessed in the presence or absence of functional TRIM5α. Before particle treatment, cells were infected with Sendai virus (SeV) expressing TRIM5 without the SPRY domain (black symbols), or an empty vector, parental Z strain of SeV (white symbols). Sixteen hours after SeV infection, cells were treated with particles for 2 hours and then infected with HIV-1-L4/5S-GFP. Representative data from six independent experiments are shown.

It is known that the intrinsic restriction factors working against HIV-1 in CM and Rh cells can be saturated by inoculation of a high dose of HIV-1 particles [19,40-42]. To determine whether alteration in the CA of HIV-1 would affect its ability to saturate restriction factors, Rh LLC-MK2 cells were pre-treated with equal amounts of VSV-G pseudotyped HIV-1 particles that were with or without SIVmac L4/5 and/or L6/7 CA to saturate intrinsic restriction factor(s). The pre-treated cells were then infected with GFP-expressing HIV-1 carrying SIVmac L4/5 CA (HIV-1-L4/5S-GFP), since we wanted to exclude any effects of CypA on the GFP expressing virus in LLC-MK2 cells. The susceptibility of particle-treated cells to virus infection was determined by the percentage of GFP-positive cells. The cells treated with the wild type (WT) particles showed greatly enhanced susceptibility to HIV-1 infection compared with non-treated cells (Fig. 4A, left), demonstrating that the intrinsic restriction factor(s) in LLC-MK2 cells were saturated by a high dose of particles. The cells treated with the particles carrying SIVmac L4/5 and those treated with particles carrying SIVmac L6/7 also showed enhanced susceptibility to HIV-1 infection (Fig. 4A, left). The cells treated with particles carrying both SIVmac L4/5 and L6/7 showed only slight enhancement of HIV-1 susceptibility (Fig. 4A, left; p = 0.007 compared by means of paired t test using all data points with the WT particle treated cells). Similarly, the cells treated with SIVmac particles showed only minor enhancement in HIV-1 susceptibility (Fig. 4A, left). Hamster TK-ts13 cells which lack TRIM5α expression, on the other hand, showed no difference in HIV-1 susceptibility among cells treated with various HIV-1 derivatives or SIVmac particles (Fig. 4A, right). As shown in Fig. 4B, similar results were obtained when we used a GFP-expressing virus with WT HIV-1 capsid (HIV-1-WT-GFP). These results indicate that both HIV-1 L4/5 and L6/7 are important for CA binding to antiviral factor(s) in Rh cells. As described previously [20], HIV-1-WT-GFP could induce infection in only small numbers of LLC-MK2 cells. In contrast, more TK-ts13 cells were infected with HIV-1-WT-GFP than with HIV-1-L4/5-GFP. It is thus possible that CypA is a supporting factor for HIV-1 replication in hamster cells as well as in human cells.

Endogenous TRIM5α seems to be a likely candidate for the antiviral factor saturated by a high dose of HIV-1 particles (Fig. 4A and 4B). To confirm this, we assessed the ability of WT and mutant HIV-1 particles to saturate the intrinsic restriction factor in the presence or absence of functional TRIM5α. The dominant negative effect of an over-expressed TRIM5 mutant lacking SPRY domain [43] was used to suppress the function of cell endogenous TRIM5α. As shown in Fig. 4C, the infection of a recombinant SeV expressing TRIM5 without the SPRY domain caused marked enhancement of HIV-1-L4/5S-GFP virus infection without prior particle treatment (crosses vs. asterisks). This indicates that this dominant negative TRIM5 mutant successfully suppressed the restriction activity of endogenous TRIM5α. Treatment with the WT HIV-1 particles also saturated the restriction factors in the cells infected with the empty vector virus (parental Z strain of SeV), while the additional effect of the dominant negative mutant TRIM5α remained unclear (Fig. 4C left, white vs. black squares). These results suggest that the intrinsic factors saturated by the WT particles were mainly endogenous TRIM5α. In contrast to the effect of the WT particle treatment, the effect of the dominant negative TRIM5 mutant on HIV-1 infection was evident when we used particles with SIVmac L4/5 and L6/7 (Fig. 4C, right, white vs. black diamonds, p = 0.007, paired t test). These findings suggest that the diminished capability of particles with SIVmac L4/5 and L6/7 to saturate restriction factors was mainly due to their loss of interaction with TRIM5α. We, therefore, concluded that the ability of HIV-1 with SIVmac L4/5 and L6/7 to bind to TRIM5α is diminished in LLC-MK2 cells.

### HIV-1 derivative with SIVmac L4/5, L6/7, and vif sequences can replicate efficiently in monkey primary cells

**Figure 5:**
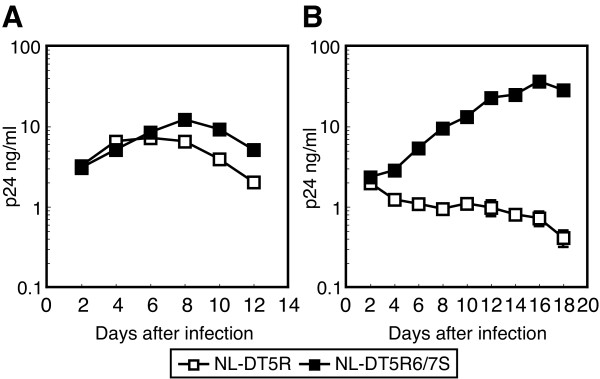
**Replication capabilities of HIV-1 derivatives in peripheral blood mononuclear cells (PBMC) from CM**. (A) PBMCs were obtained from CM, after which the CD8+ cells were removed, and the cells were stimulated with PHA-L for 1 day. (B) CD8-depleted CM PBMC were first stimulated with 1 μg/ml of PHA-L for 2 days and then with human IL2 100 U/ml for 2 more days. Equal amounts of p24 of NL-DT5R (white squares) or NL-DT5R6/7S (black squares) were inoculated, and the culture supernatants were collected periodically. p24 antigen levels were measured by ELISA. Values represent means with actual fluctuations of duplicate samples added. The values for mock infected cell culture supernatants were zero in the ELISA assay.

To verify the effect of additional replacement of HIV-1 L6/7 with that of SIVmac in primary CM cells, we prepared PBMCs from CM and removed CD8+ cells by means of magnetic beads. The cells were then stimulated for 1 day with 1 μg/ml of PHA-L. NL-DT5R6/7S showed more efficient replication than did the parental NL-DT5R in these cells and reached its peak titer 8 days after infection (Fig. 5A). For prolonged stimulation, CD8-depleted CM PBMCs were first stimulated with 1 μg/ml of PHA-L for 2 days and then with human IL2 100 U/ml for 2 more days. In these cells, NL-DT5R with HIV-1 L6/7 did not grow at all. On the other hand, NL-DT5R with SIVmac L6/7 (NL-DT5R6/7S) grew in CM primary cells in response to prolonged stimulation by PHA and IL-2 to reach titers, similar to those attained in cells with short stimulation, up to 8 days after infection (Fig. 5A and 5B). Furthermore, NL-DT5R6/7S continued to grow to much higher titers and reached its peak titer 16 days after infection; this higher peak may be due to better proliferation of these cells than those cells receiving short term stimulation (Fig. 5B). These results confirmed that the replicative capability of HIV-1 in CM cells was augmented by the additional replacement of L6/7 of CA with the corresponding sequence from SIVmac.

## Discussion

We created simian-tropic HIV-1 with more efficient replication capability in CM cells using the knowledge obtained from our previous study of TRIM5α and HIV-2 capsid sequence variations [32] Introduction of the entire SIVmac L6/7 CA into the previously constructed version of HIV-1 derivatives containing SIVmac L4/5 CA and *vif *[21] caused only a four amino acid change in CA but showed improved replication capability of HIV-1 in the CM cell line HSC-F. Introduction of the entire SIVmac L6/7 CA into NL-DT5R, which has two additional amino acid mutations in the *env *gene, enhanced replication in CD8+ cells-depleted CM PBMCs. After prolonged stimulation of CM PBMCs, replication of the original version of NL-DT5R was suppressed while that of NL-DT5R with SIVmac L6/7 was not. It would thus be of interest to test whether those HIV-1 derivatives with both L4/5 andL6/7 from SIVmac can induce infection of CM *in vivo*.

While the high-dose inoculation of WT HIV-1 particles into Rh cells saturated endogenous TRIM5α and enhanced subsequent infection with HIV-1, the introduction of HIV-1 particles that contained both L4/5 and L6/7 from SIVmac greatly impaired the ability of the particles to saturate TRIM5α. When we replaced either HIV-1 L4/5 or L6/7 with the corresponding sequence from SIVmac, these particles still saturated TRIM5α. These findings suggest that TRIM5α recognized the overall structure composed of both L4/5 and L6/7 of HIV-1 CA. Our previous results from computational 3D-structure modeling analysis of HIV-2 CA support this hypothesis [32]. The 120th amino acid of HIV-2 CA, which affects viral susceptibility to TRIM5α restriction, was located in L6/7. It is especially worth noting that the amino acid substitution at the 120th position was previously predicted to induce marked changes in the configuration of L6/7 and the L6/7 with the CM TRIM5α-sensitive Pro positioned most closely to L4/5 of HIV-2 [32]. It would, therefore, be interesting to investigate whether monkey TRIM5α proteins recognize CypA bound-L4/5 of HIV-1 CA.

During the preparation of our manuscript, Lin and Emerman reported that SIVagmTAN with both HIV-1 L4/5 and L6/7 was susceptible to Rh-TRIM5α restriction [44]. Our result is consistent with their finding, since the HIV-1 particles with both SIVmac L6/7 and SIVmac L4/5 showed reduced saturation activity for TRIM5α in Rh cells compared with HIV-1 particles with SIVmac L4/5 alone. Hatziioannou et al. very recently reported that stHIV-1 strains, which differ from HIV-1 only in the *vif *gene, could efficiently replicate in pig-tailed monkey and proposed a pig-tail monkey model of HIV-1 infection [45]. This is not surprising, since pig-tailed monkeys lack a TRIM5α protein, and the dominant form of TRIM5 expressed in this monkey species is a TRIMCyp fusion protein lacking anti-HIV-1 activity [46-48].

When we subjected CD8-depleted CM PBMC to prolonged stimulation, NL-DT5R6/7S grew efficiently but NL-DT5R did not. Since the expression levels of TRIM5α mRNA in human PBMC increased after stimulation with PHA and IL2 for 3 days (data not shown), we speculated that the higher expression levels of CM-TRIM5α in fully stimulated CM cells resulted in efficient restriction of NL-DT5R. However, no clear enhancement of CM TRIM5α mRNA expression could be detected in the CM cells subjected to prolonged stimulation (data not shown). The reason why NL-DT5R failed to grow in CM cells with prolonged stimulation is not yet clear, but it is possible that fully stimulated CM cells exerted stronger intrinsic inhibitory activity against HIV-1 infection than those with short-term stimulation.

NL-DT5R6/7S and NL-ScaVR6/7S replicated less efficiently in human MT4 cells than did the parental NL-DT5R and NL-ScaVR. One possible explanation is that the virus with SIVmac L6/7 became resistant to CM TRIM5α but became more sensitive to human TRIM5α, since the latter can restrict SIVmac more efficiently than HIV-1. Another possibility is that replacement of CA allowed the virus to evade the intrinsic inhibitory factors in CM cells but impaired viral replication *per se*.

We used the CM T cell line HSC-F and CD8+ cell-depleted PBMC from CM but not from Rh for our replication experiments. Although we observed an improvement of viral replication in CM cells, we cannot assume that the replacement of L4/5 and L6/7 is enough for HIV-1 to replicate to high titers in Rh cells since the CM TRIM5α resistant HIV-2 mutant virus GH123 (Q) was found to be restricted by Rh TRIM5α [34]. NL-DT5R6/7S and NL-ScaVR6/7S also showed less efficient replication capability than did SIVmac (Fig. 1). We are currently trying to adapt these viruses to CM and Rh cells by means of long-term passaging in the hope of introducing compensating mutations that can overcome these disadvantages and further augment their replicative capabilities in human and simian cells to reach a similar level as seen with SIVmac.

## Conclusion

We have succeeded in improving simian-tropic HIV-1 for more efficient replication in CM cells by introduction of the SIVmac L6/7 CA sequence. It will be of interest to determine whether the HIV-1 derivatives with SIVmac L4/5 and L6/7 can induce infection in cynomolgus monkeys *in vivo*. Even if they fail to do so, further modification and/or adaptation of the current version of simian-tropic HIV-1 in monkey cells might be expected to lead to the development of an HIV-1 infection model in OWMs. This model has been long-awaited as a tool for vaccine development and as a model for better understanding of AIDS pathogenesis.

## Abbreviations

OWM: old world monkey; CM: cynomolgus monkey; Rh: rhesus monkey; SHIV: HIV-1/SIV chimeric virus; CypA: cyclophilin A; TRIM: tripartite motif; CA: capsid; PBMC: peripheral blood mononuclear cell; GFP: green fluorescence protein; VSV-G: vesicular stomatitis virus glycoprotein; SeV: Sendai virus; L4/5: a loop between α-helices 4 and 5; L6/7: a loop between α-helices 6 and 7.

## Competing interests

The authors declare that they have no competing interests.

## Authors' contributions

TS and EEN designed the research, AK, AS, YS, and EEN performed the research, TS, MN, AA, and EEN analyzed the data, and AA, HA, TS, and EEN wrote the paper.
